# Retraction Note: Management of uterine leiomyoma and adenomyosis: role of hysteroscopy in diagnosis and norethindrone in the treatment

**DOI:** 10.1038/s41598-026-43159-7

**Published:** 2026-03-09

**Authors:** Ahmed Ali, Alaaeldin Youssef, Ali El Saman, Hisham Abou Taleb

**Affiliations:** Department of Obstetrics and Gynecology, Faculty of Medicine, Assiut University, Asyut, 17515 Egypt

Retraction of: *Scientific Reports* 10.1038/s41598-025-28540-2, published 03 December 2025

The Editors have retracted this Article.

After publication, concerns have been raised regarding the data, specifically that some of the reported errors appear to be highly unlikely given the reported means. In addition, some of the reported results appear not to make sense arithmetically.

The Editors requested the raw data underlying the study and the ethical approval documentation, but the Authors did not provide the data and the ethical documentation provided did not address the concerns related to ethical oversight. The Editors have therefore lost confidence in the results and conclusions of this Article.

None of the Authors replied to the correspondence from the Editors about this retraction.

